# Supplementary material for: Risk factors associated with the discordance in kidney function decline rate in identical twins
Source: PLoS One. 2023 Apr 13;18(4):e0284154. doi: 10.1371/journal.pone.0284154 (PMC10101412; doi:10.1371/journal.pone.0284154)
Supplement: Attachment — Submitted filename: Abstract.docx [file pone.0284154.s001.docx]

**Minor Revision**

**Abstract,**

*Background*

Line 26:

Line 27 and 28: “We aim to analyze the effect of environmental factors on kidney dysfunction in an identical twin”- This is not the aim of this study. I suggest it is replaced with “This study set out to identify the risk factors for high discordance in kidney function decline in an identical twin cohort.

*Methods:*

lines 29-31: According to this manuscript, the 333 identical twins were categorized into two groups according to the inter-twin difference in the annual eGFR decline: High discordance twins and low discordance twins, and individual twin were further classified as either a slow progressor or a rapid progressor.

Line 30 : “ eGFR” should be written in full as “estimated glomerular filtration rate (eGFR)”. It can be abbreviated subsequently

Lines	Please kindly remove the sentence “identical twins with diabetes were excluded”. This sentence should have come before “The mean difference in the annual …………. who had >5%.........annual eGFR decline.”

**Introduction:**

Line 1-2:

Lines 48-49 : consider changing the phrase “ but most patients with CKD have an elusive etiology.” to “ however, some patients with CKD have an elusive etiology.”

Line 51

Line 59-61: Please consider changing the phrase “non genetic factors affecting kidney dysfunction besides natural aging in the general population using an identical twin cohort to exclude genetic contribution” to “non genetic factors associated with annual kidney function decline in the general population besides natural aging using an identical twin cohort.”

**Materials and Methods:**

*Study Cohort:*

Line 69: Please consider changing “can be found elsewhere “to “have been published elsewhere”

Line 71: twins with background kidney disease should also be excluded from the cohort.

*Study Outcome*

The study outcomes are not clearly stated in this paragraph. The authors should let us know what their study outcomes are.

*Data Collection:*

This section should come immediately after Study cohort (Before study outcome.)

Lines 96 and 99 : I suggest the authors replace the word ‘collected’ with “retrieved”

Lines 96/97: Pls kindly remove the sentence “ Body fat percentage was measured by dual X ray absorptiometry”

**Statistical Analysis:**

Lines 109 and 110: Please remove phrase “to determine whether the mean difference between pairs of measurement was zero or not”.

Lines 111-112: If level of significance was set at 0.05, why were significant variables with (p<0.1) in univariate analysis adjusted for in multivariate analysis ? If level of significance for this study was set at 0.05, please kindly stick to it in your analysis.

**Results**

*Baseline characteristics and annual eGFR change in twins*

Line 122 &123: The sentence “We calculated the intra-twin differences in the annual eGFR change in the slow progressor group minus that in the fast progressor group.” Should come under Methods section.

Line 128&129: the subtitle “Difference of clinical characteristics of renal function between the twins in slow or rapid progressor groups- should be changed to “ Baseline Characteristics of the Low and high discordance twins stratified by the rate of annual kidney function decline as either a low progressor or rapid progressor).

Line 130-132: The sentence “To find the factors affecting the intra twin difference in eGFR decline rates, the twins were classified as high discordance and low discordance twins who had >5% and <5% of intra twin difference in annual eGFR change respectively” should have been mentioned under “ study outcome in Materials and Methods”

Line 144: It should be “Risk analysis for high discordance in eGFR decline in twins ”

Line 145-146. The sentences” To find the risk factors associated with high discordance ………….were conducted” and “ Except for age and sex, all baseline characteristics were included as covariates in the form of intra twin differences.” Should be under “S tudy Outcome in Materials and Methods”. These sentences should not be under the “Result section”

Lines 156-160: Fig 2. Blood hemoglobin levels according to the difference in eGFR decline in Identical Twins- should be the title for Fig. 2 while “With dotted line as the center, the plot
on the left ………………low and high groups respectively. Circular dots indicate the slow progressor……. whereas the triangular dots indicate the rapid progressor. The lines connecting the circular and triangular dots represent each pair of twins” should be the legends to Fig 2.

Discussion:

Lines 183-186: The authors stated that they (their study) found that chronic illness and nutritional insufficiency might play a significant role in rapid kidney function decline in the general population.

This study did not demonstrate the above findings. Authors may need to re phrase this sentence.

Fig 1

Twins in KoGES twin and Family cohorts (n=423 pairs)

Exclusion Criteria:

1. Fraternal twins (n=71 pairs)
2. One or both twins had diabetes (n = 17 pairs
3. Error in date of birth (n =2 pairs)

Included identical twins

N = 33 pairs)

Annual eGFR decline	Annual eGFR decline < 5%

≥ 5%

High Discordance group (n = 51 pairs)

Low Discordance group (n=282 pairs)

Slow Progressor (n = 51)

Slow Progressors (n =282)

Rapid Progressor (n = 51)

Rapid Progressor (n =282)

Major Revision:

Jeong Ah Hwang et al in this study assessed for the risk factors associated with high discordance in annual kidney function decline

in a cohort of identical twins. Though, this is the first study to look at factors associated with discordance in kidney function decline in a cohort of identical twins, this article is very difficult to comprehend. In addition, there are lot of issues with the Materials and method section of this article.

- Authors only excluded diabetic and those who had error in data sets. One is not sure if the cohort they studied have some individuals with background chronic kidney disease. I there are, they need to be excluded from the study. It will add value, if in the result section, the mean(±SD) baseline eGFR of the cohort could be displayed in Table 1.

- The authors did not clearly state what their study outcomes were under the Study outcome section.

- Authors did not clearly define the terms “Rapid progressors and Slow progressors”. Definition of slow/rapid progressors for this study is not clear.
