# Supplementary material for: Risk factors associated with the discordance in kidney function decline rate in identical twins
Source: PLoS One. 2023 Apr 13;18(4):e0284154. doi: 10.1371/journal.pone.0284154 (PMC10101412; doi:10.1371/journal.pone.0284154)
Supplement: Attachment — Submitted filename: plos_one_response_final.docx [file pone.0284154.s002.docx]

PONE-D-22-15736

Risk factors associated with the discordance in kidney function decline rate in identical twins

**Dear Editors and Reviewer,**

**We are grateful for your time in reviewing our manuscript. Please check our point-by-point responses. Changes made in our revised manuscript are in coloured text with track change.**

PLOS ONE

Additional Editor Comments:

Ji Eun Kim and colleagues conducted a retrospective identical twins cohort study to assess the risk factors associated with the discordance in kidney function decline rate in identical twins. They found that blood hemoglobin level may play a role in the individual differences in kidney function decline. However, the authors should take into account the following in order to improve the quality of the manuscript.

Major comments:

1. There are concerns about the absence of details in the methodology adopted and in the lack of clarity of some definitions. It is very important for the authors to confirm if participants with background CKD were excluded or not? It is not clear why the authors did not assess for urinary protein excretion rate (proteinuria)? I suggest that the result of estimation of urinary protein excretion rate should be provided. Also, it is important for the authors to define slow progressors and rapid progressors using the 2012 KDIGO Clinical Practice guideline.

**Response:** Thank you for your valuable comments. In the original article, we included patients who had CKD. According to the editor and reviewer’s comment, we checked the baseline eGFR to exclude CKD patients defined with eGFR under 60 mL/min/1.73m2 in this cohort. Thus, only one pair of twins was excluded. The presence of proteinuria was not examined because urinalysis, random urine protein, or 24h urine collection were not performed. Therefore, in the Limitation section, we included it in the list. Furthermore, based on your suggestion, the criteria for calculating CKD progression was changed from percent to absolute change of eGFR of >5 mL/min/1.73m2 /yr. The changes can be found in our revised manuscript. (Page 5, lines 70 to 73; Page 6, lines 95 to 104)

2. Exclusion criteria – How many of this cohort had heart failure and glomerulonephritis? Were these clinical entity excluded?

**Response:** We appreaciate your question. Unfortunately, the clinical information on heart failure and glomerulonephritis was unavailable in this cohort. Thus, we included this as one of the limitations in the Discussion section. (Page 15, lines 270 to 272)

3. How was acute deterioration of kidney function excluded in the study participants?

**Response:** Many thanks for your helpful question. The presence of absence of acute kidney deterioration cannot be assessed because there was only two-point sampling data for almost 3 years (median 38.6 months), apart in this cohort. The sampling frequency problem was also described in the Limitation section. (Page 15, lines 269 to 270)

4. The sentence “We calculated the intra-twin difference in the annual eGFR change by calculating the eGFR change in the slow progressor group minus that in the fast progressor group.” (line 122 – 123) should be moved to section on methodology.

**Response:** Thank you for your suggestion. Based on this, thi sentences was included in the Methodology section.

5. The authors should state the baseline eGFR for the study cohort.

**Response:** We are grateful for your review. Based on this, we added the information on eGFR baseline (Table 1). (Page 8) .

6. How was hypertension defined in this cohort? I wonder if the rate of CKD progression is related to hypertension, so would be interested in how many slow progressor vs rapid progressor had hypertension. The percentage of slow progressors vs rapid progressors with hypertension should be included in table 1.

**Response:** Many thanks for pointing this out. Hypertension was defined through the responses in the questionnaire. Participants in this study answered (yes or no) to questions whether diagnosed with hypertension or taking antihypertensive medications. The proportion of patients with hypertension was provided in Table 1.

7. It is not very clear how the authors arrived at the choice and numbers of independent variables used in the multivariate logistic regression model. I suggest that the regression model should be re-analysed with the inclusion of established risk factors for CKD progression (e.g hypertension and proteinuria). Also the method (backward or forward or stepwise) of inclusion of independent variables in the model should be stated.

**Response:** We are grateful for your valuable suggestions. As I responded in previous comment #1, proteinuria was not available in this cohort. Therefore, we added hypertension as a risk factor for re-analysis alone. In the revised model, we used age, sex, hypertension, and those factors significant in the univariable analysis as covariables for multivariable logistic regression. The stepwise method for variable selection was not used in this study because it only includes covariates with small p-values (as in stepwise regression), which may cause overestimated coefficients that are more likely to be selected. Thus, it may cause bias from the null hypothesis [1,2].

References

1.	Rothman KJ, Greenland S, Lash TL. Modern epidemiology. 3rd ed., thoroughly rev. and updated ed. Philadelphia: Wolters Kluwer Health/Lippincott Williams & Wilkins; 2008.

2.	Lydersen S. Statistical review: Frequently given comments. Ann Rheum Dis. 2015;74: 323–325. doi: [10.1136/annrheumdis-2014-206186](https://doi.org/10.1136/annrheumdis-2014-206186).

8. What were the rates of missing data? If data are complete for all participants, this should be stated.

**Response:** Thank you for pointing this out. The rate of missing data is provided in Table R1. There was no variable with a missing rate >5% and those were handled by “complete case analysis” (disregarding cases with partially missing data).

In addition, the missing data handling in the statistical analysis paragraph was also added in the Methods section.

| Table R1. The rate of missing values | |  |
| --- | --- | --- |
|  | Slow progressor N=332 | Rapid progressor N=332 |
| Annual eGFR change, % | 0 (0%) | 0 (0%) |
| Demographic characteristics | |  |
| Age, year | 0 (0%) | 0 (0%) |
| Male sex, n(%) | 0 (0%) | 0 (0%) |
| Body mass index, kg/m2 | 0 (0%) | 0 (0%) |
| Waist to hip ratio, cm/cm | 1 (0.3%) | 0 (0%) |
| Body fat percentage, % | 4 (1.2%) | 5 (1.5%) |
| Smoking status, n(%) | 0 (0%) | 1 (0.3%) |
| Alcohol cumsumption, n(%) | 1 (0.3%) | 2 (0.6%) |
| Systolic blood pressure, mmHg | 1 (0.3%) | 2 (0.6%) |
| Dyastolic blood pressure, mmHg | 1 (0.3%) | 2 (0.6%) |
| Clinical histories |  |  |
| Myocardial infarction, n(%) | 0 (0%) | 1 (0.3%) |
| cerebral infarction, n(%) | 0 (0%) | 1 (0.3%) |
| Hypertension, n(%) | 0 (0%) | 1 (0.3%) |
| Dyslipidemia, n(%) | 0 (0%) | 1 (0.3%) |
| Laboratory findings |  |  |
| Baseline eGFR, mL/min/1.73m2 | 0 (0%) | 0 (0%) |
| Hemoglobin, g/dL | 0 (0%) | 0 (0%) |
| Glucose, mg/dL | 0 (0%) | 0 (0%) |
| Albumin, g/dL | 0 (0%) | 0 (0%) |
| Total cholesterol, mg/dL | 0 (0%) | 0 (0%) |
| LDL cholesterol, mg/dL | 0 (0%) | 0 (0%) |
| Triglyceride, mg/dL | 0 (0%) | 0 (0%) |
| Uric acid, mg/dL | 1 (0.3%) | 1 (0.3%) |
| Nutrition intake (body weight adjusted) | |  |
| Total calorie intake, kcal | 10 (3.0%) | 10 (3.0%) |
| Protein, g | 10 (3.0%) | 10 (3.0%) |
| Fat, g | 10 (3.0%) | 10 (3.0%) |
| Sugar, g | 10 (3.0%) | 10 (3.0%) |
| Ca, mg | 10 (3.0%) | 10 (3.0%) |
| P, mg | 10 (3.0%) | 10 (3.0%) |
| Fe, mg | 10 (3.0%) | 10 (3.0%) |
| K, mg | 10 (3.0%) | 10 (3.0%) |
| Na, mg | 10 (3.0%) | 10 (3.0%) |

9. Line 136-138 “in the high discordance group, the slow progressor group showed a higher …………..serum fasting glucose ……..than those of the rapid progressor group” It will be nice to see median (interquartile range) value for serum fasting glucose.

**Response:** Thank you for your valuable comment. Based on this, we changed the glucose value in Table 1 from mean with standard deviation to median with interquartile range. (Page 8)

10. Table 1 – The mean±SD values stated for albumin and iron for slow progressor group and rapid progressor group in both low and high discordance twins were exactly the same but the p-values for the 2 pairs were different. I suggest that the authors should double check their data and statistics.

**Response:** Thank you for pointing this out, which helped our manuscript to become better. Therefore, we reanalyzed all group comparisons between low and high discordance groups after excluding one pair of twins due to low baseline eGFR, The mean values and statistics for variables were all changed (Table 1), and data were carefully checked.

11. Inability to assess the relationship between gut microbiota dysbiosis, nutrient absorption and decline in eGFR should be discussed as part of the limitation.

**Response:** We appreciate your valuable insight. Based on this, the further need for gut microbiota and nutrition study was included in the Limitation section:

“Though we assessed the food consumption data in this cohort, other nutritional factors, including gut microbiota and their metabolic function which may be related to the environmental difference in twins, need further evaluation.” (Page 15, lines 266 to 269)

12. The conclusion did not reflect the exact finding of this study – nutritional related factors were not established as predictors of high discordance of eGFR decline. I suggest a revision to the concluding statement.

We changed the concluding statement. Thanks for helpful comment.

Minor comments:

1. No page numbers

**Response:** Thank you for your careful review. Page numbers were added on our revised manuscript.

2. There are typos that require correction.

**Response:** We appreciate that you noticed them. A professional native speaker checked this revised manuscript.

Reviewers' comments:

Reviewer's Responses to Questions

**Comments to the Author**

Reviewer #1: Thank you for inviting me to review this manuscript “Risk factors associated with the discordance in kidney function decline rate in identical twins.” The authors aimed to analyze the effect of environmental factors on kidney dysfunction in an identical twin cohort.

I have the following concerns with the study.

Dear Reviewer,

We are grateful for your time in reviewing our manuscript. Please check our point-by-point responses. Changes made in our revised manuscript are in red text.

1. Assessment of kidney function decline takes into account absolute rate of loss of kidney function (Al-Aly Z, Zeringue A, Fu J et al. Rate of kidney function decline associates with mortality. J Am Soc Nephrol 2010; 21: 1961–1969) or percentage change in the kidney function (Cheng TY, Wen SF, Astor BC et al. Mortality risks for all causes and cardiovascular diseases and reduced GFR in a middle-aged working population in Taiwan. Am J Kidney Dis 2008; 52: 1051–1060).

**Response:**Thank you for the valuable comment. In our original manuscript, we used percent change of kidney function for defining rapid progression or high discordance of kidney function. However, we changed the classification criteria by an absolute rate of loss of kidney function, which is used for rapid progression definition in the KDIGO guideline based on the reviewers’ comments and suggestions [1].

Reference

1. Kidney Disease: Improving Global Outcomes (KDIGO) CKD-MBD Work Group. "KDIGO clinical practice guideline for the diagnosis, evaluation, prevention, and treatment of Chronic Kidney Disease-Mineral and Bone Disorder (CKD-MBD)." Kidney international. Supplement 76.113 (2009): S1-130.

2. The authors categorised participants into high discordant and low discordant and then into rapid progressors and slow progressors in each group based on ≥5% annual eGFR changes. The KDIGO 2012 clinical practice guideline defined progression as a “certain drop” in eGFR defined as a drop in GFR grade associated with a ≥25% decline in eGFR from baseline and “Rapid Progression” as sustained decline in eGFR of more than 5ml/min/1.73m2/yr. I suggest that authors should use clear guidelines in defining technical terms such as rapid progressors.

**Response:** Many thanks for pointing this out. Based on this, we changed the definition of kidney dysfunction in this study with an absolute rate of eGFR decline. We categorized the twins by difference of eGFR decline rate >5 mL/min/1.73m2 /yr (high discordance) or ≤ 5 mL/min/1.73m2 /yr (low discordance). The number of twins in the high discordance group does not change despite changing the definition.

3. Authors should kindly provide detailed information on the number of times samples were taken to assess renal function during the 3 years period. This is important because studies have shown that the more the frequency of samples collection, the better the confidence and precision in assessing progression of kidney function. Furthermore, do the rapid progressors have a sustained annual eGFR decline of at least 5ml/min/1.73m2/yr

**Response:** We appreciate your valuable insights. Unfortunately, in this “KoGES twin and family cohort”, samples were taken on two-time point alone. As a result, the median duration between the first sampling (baseline) and 2^nd^ sampling (follow-up) was 38.6 [28.4–49.7] months. The lack of multiple sampling for each patient was included in the Limitation section of our revised manuscript. (Page 15, lines 269 to 271)

4. Urine albumin excretion estimation such as Urine Albumin-creatinine ratio (ACR) is a recognized predictor of progression of kidney disease. Since this or any other data showing evidence of proteinuria was not included in this study, I suggest it should be included as a limitation.

**Response:** Thank you for your helpful suggestion. However, in this cohort, only blood sample measurements were available and urinary measurement was not included. Therefore, we included the lack of urinalysis as one of the limitations. (Page 15, line 270)

5. Other predictors of kidney function decline should be included in the multiple logistic regression model with the exception of diabetes since its an exclusion factor. I suggest that in addition to Age and gender, recognized predictors of decline in kidney function must be chosen apriori based on previous literature.

**Response:** We appreciate your comment. We included multiple covariates, such as body mass index, body fat percentage, hypertension, baseline estimated glomerular filtration rate, hemoglobin, total cholesterol, glucose, triglyceride, uric acid, age, and gender for multivariable analysis, based on significance in univariate analyses. The incidence of other clinical histories, such as myocardial infarction and cerebral infarction, was too low to include as a covariate in this twin cohort.

6. I have a strong concern about how the independent variables in the multivariable logistic regression model were derived (i.e difference between slow progressors and rapid progressors). This may have resulted in loss of valuable information during the regression analysis. Also, authors need to demonstrate how they checked for Multicollinearity and other assumptions of Multiple logistic regression model especially for variables like body mass index and body fat percentage. I suggest the authors should use Variance inflation factor. Kindly show the overall statistical details of your Multiple Logistic regression model.

**Response:** Thank you for pointing this out. We performed a regression model with variance inflation factor (VIF) analysis for multicollinearity check with a multivariable model, and we found the maximum score of VIF was 1.67, sufficient to proceed to analysis. The table about VIF scores for each variable is provided below.

Table R2. The result of variance inflation factor analysis with variables included in multivariable analysis

| Variable | VIF | 1/VIF |
| --- | --- | --- |
| BMI | 1.67 | 0.599714 |
| Body fat percentage | 1.47 | 0.680838 |
| Uric acid | 1.39 | 0.721975 |
| Triglyceride | 1.26 | 0.795747 |
| Glucose | 1.21 | 0.827063 |
| baseline eGFR | 1.16 | 0.860098 |
| Total cholesterol | 1.16 | 0.864071 |
| Hemoglobin | 1.13 | 0.888805 |
| Hypertension | 1.07 | 0.935191 |
| Sex | 1.04 | 0.962675 |
| Age | 1.01 | 0.989445 |
| Mean VIF | 1.23 |  |

7. Kindly demonstrate evidence of adequacy of sample size.

**Response:** Thank you for your suggestion. In this study, the sample size can be calculated for logistic regression with two-tails. The mean hemoglobin difference between twins was 0.11, with a standard deviation of 1.06. Pr(Y=1|X=1) H0 was calculated as 0.314 (16 events occurred outside one standard deviation). R-squared was calculated as 0.0827 with other variables. Significance levels for alpha error probability and power were set at 0.05 and 0.8, respectively. Then, we got a total sample size of 304, which is smaller than the actual sample size we used (n=332).

Since there was no previous study similar to this, we conducted the study on the entire cohort. However, post hoc sample size analysis showed that the number of samples used in this study was not inappropriate**.**

8. I suggest that Hypoxia inducible factors (HIF) including oxygen-dependent and oxygen-independent HIF-regulators should be discussed in relation to haemoglobin level which is the only independent predictor.

**Response:** Thank you for your helpful suggestion. Based on this, the result has been changed to low glucose and low hemoglobin levels as risk factors for high discordance because of the change in the number of study population. Therefore, we added brief information on the potential pathophysiology involved in anemia and CKD progression in the Discussion section.

“The mechanisms underlying the association between anemia and CKD progression are unclear. However, it has been hypothesized that anemia causes tissue hypoxia and consequently leads to alterations in gene expression patterns, triggering adaptive pathways related to hypoxia-inducing factors in parallel with the induction of noxious mediators involved in the progression of renal injury.” (Page 14, lines 244 to 248)

9. Figure 1 should be expanded to provide clarity in the study design. This should show clearly the number of patients in High discordant group and low discordant group. Also,it should clearly show the number of participants in slow progressor and rapid progressor.

**Response:** Many thanks for pointing this out. For the clarity of our study design, we added a new figure in our revised manuscript (Figure 2), including group design and the number of twins in each group. (Page 106)

Reviewer #2: Major Revision:

Jeong Ah Hwang et al in this study assessed for the risk factors associated with high discordance in annual kidney function decline
in a cohort of identical twins. Though, this is the first study to look at factors associated with discordance in kidney function decline in a cohort of identical twins, this article is very difficult to comprehend. In addition, there are lot of issues with the Materials and method section of this article.

- Authors only excluded diabetic and those who had error in data sets. One is not sure if the cohort they studied have some individuals with background chronic kidney disease. I there are, they need to be excluded from the study. It will add value, if in the result section, the mean(±SD) baseline eGFR of the cohort could be displayed in Table 1.

**Response:** Thank you for your careful review. However, we did not check the baseline eGFR in our original manuscript, and we totally agree with your concern; therefore, we reanalyze the cohort to exclude baseline eGFR <60 mL/min/1.73m^2^. We found only one person who had eGFR <60 mL/min/1.73m^2^. Hence, we excluded one pair of twins (one of them had a low eGFR) for reevaluation (the number of total twins turned 332 pairs). In addition, we included the baseline eGFR value in Table 1.

- The authors did not clearly state what their study outcomes were under the Study outcome section.

**Response:** Thank you for pointing this out. Based on this, the study outcome section was modified to clarify the study outcome.

*“Study outcome*

This study aimed to determine the non-genetic risk factor which predicts the rapid progression of kidney function. Therefore, the main outcome of this study is the risk for high discordance twins who have a disparity of eGFR decline rate of >5 mL/min/1.73m^2^ /yr. The association between outcome (high discordance group) and various clinical and demographic characteristics was assessed.” (Page 6, lines 108 to 112)

- Authors did not clearly define the terms “Rapid progressors and Slow progressors”. Definition of slow/rapid progressors for this study is not clear.

**Response:** Many thanks for your expert review. Based on this, we rephrase and clarify the terms “Rapid progressors and Slow progressors” in the study group classification section. Moreover, we added Figure 2 to describe the study method in a simple way.

Minor Revision

Abstract,

Background

Line 26:

Line 27 and 28: “We aim to analyze the effect of environmental factors on kidney dysfunction in an identical twin”- This is not the aim of this study. I suggest it is replaced with “**This study set out to identify the risk factors for high discordance in kidney function decline in an identical twin cohort.**

**Response:** Thank youfor the correction. Based on this, we changed the manuscript with suggested sentence.

Methods:

lines 29-31: According to this manuscript, the 333 identical twins were categorized into two groups according to the inter-twin difference in the annual eGFR decline: High discordance twins and low discordance twins, and individual twin were further classified as either a slow progressor or a rapid progressor.

Line 30 : “ eGFR” should be written in full as “**estimated glomerular filtration rate (eGFR**)”. It can be abbreviated subsequently

Lines Please kindly remove the sentence “identical twins with diabetes were excluded”. **This sentence should have come before “The mean difference in the annual …………. who had >5%.........annual eGFR decline.”**

**Response:** Thank you for pointing this out. We have now changed the placement of the abbreviation list and the sentence was moved at the end of the paragraph. The sentence has also been changed because we reanalyzed the data after excluding baseline eGFR <60 mL/min/1.73m^2^. (Page 3, lines 28 to 34)

Introduction:

Line 1-2:

Lines 48-49 : consider changing the phrase “ but most patients with CKD have an elusive etiology.” to “ **however, some patients with CKD have an elusive etiology.**”

Line 51

Line 59-61: Please consider changing the phrase “non genetic factors affecting kidney dysfunction besides natural aging in the general population using an identical twin cohort to exclude genetic contribution” to “**non genetic factors associated with annual kidney function decline in the general population besides natural aging using an identical twin cohort**.”

**Response:** Thank you for your great suggestions. Therefore, we changed the sentence based on your recommendation.

Materials and Methods:

Study Cohort:

Line 69: Please consider changing “can be found elsewhere “to “**have been published elsewhere**”

**Response:**Many thanks. We changed the sentence following your suggestion.

Line 71: twins with background kidney disease should also be excluded from the cohort.

**Response:** We excluded the twins who have baseline eGFR under 60 ml/min/1.73m^2^, as in response for your major revision. However, other kidney disease cannot be estimated and excluded because of the lack of urinarlysis and other findings such as kidney biopsy. We stated this as limitation in discussion section.

Study Outcome

The study outcomes are not clearly stated in this paragraph. The authors should let us know what their study outcomes are.

**Response:** Thank you. The study outcome paragraph to clarify the objective of this study was changed.

Data Collection:

**This section should come immediately after Study cohort** (Before study outcome.)

**Response:** Thank you. Based on your recommendation., we changed the order of paragraph.

Lines 96 and 99 : I suggest the authors replace the word ‘collected’ with **“retrieved**”

Lines 96/97: Pls kindly **remove the sente**nce “ Body fat percentage was measured by dual X ray absorptiometry”

±

**Response:** We are grateful for the expert review. Therefore, we replaced the word ‘collected’ with ‘retrieved’ and removed the sentence “ Body fat percentage was measured by dual X ray absorptiometry.”

Statistical Analysis:

Lines 109 and 110: Please **remove phrase** “to determine whether the mean difference between pairs of measurement was zero or not”.

**Response**: Thank you. We removed the phrase according to your suggestion.

Lines 111-112: If level of significance was set at 0.05, why were significant variables with (p<0.1) in univariate analysis adjusted for in multivariate analysis ? If level of significance for this study was set at 0.05, please kindly stick to it in your analysis.

**Response:** Thank you for pointing this out. According to your recommendation, we changed the covariables included for multivariable analysis as variables, which showed p < 0.05 in univariable analysis. Thus, serum cholesterol was removed from covariables.

Results

Baseline characteristics and annual eGFR change in twins

Line 122 &123: The sentence “**We calculated the intra-twin differences in the annual eGFR change in the slow progressor group minus that in the fast progressor group.” Should come under Methods section.**

**Response:** Thank you. based on this, the sentence has been deleted to avoid redundancy.

Line 128&129: the subtitle “Difference of clinical characteristics of renal function between the twins in slow or rapid progressor groups- should be changed to “ Baseline Characteristics of the Low and high discordance twins stratified by the rate of annual kidney function decline as either a low progressor or rapid progressor).

**Response:** Thanks for the correction. We changed the subtitle as you recommended.

Line 130-132: The sentence “To find the factors affecting the intra twin difference in eGFR decline rates, the twins were classified as high discordance and low discordance twins who had >5% and <5% of intra twin difference in annual eGFR change respectively” should have been **mentioned under “ study outcome in Materials and Methods”**

**Response:** Many thanks. This sentence was also deleted because a similar sentence could be found in the method part.

Line 144: It should be “**Risk analysis for high discordance in eGFR decline in twins** ”

**Reponse:** Thanks for pointing out the grammatical error. This subtitle has been corrected according to your advice.

Line 145-146. The sentences” To find the risk factors associated with high discordance ………….were conducted” and “ Except for age and sex, all baseline characteristics were included as covariates in the form of intra twin differences.” **Should be under “S tudy Outcome in Materials and Methods”.** These sentences should not be under the “Result section”

**Response:** We appreciate your careful review. Based on this, we moved and edited the sentences to the statistical analysis paragraph in Materials and Methods section and removed then from the Results section.

Lines 156-160: Fig 2. Blood hemoglobin levels according to the difference in eGFR decline in Identical Twins- should be the title for Fig. 2 while “With dotted line as the center, the plot
on the left ………………low and high groups respectively. Circular dots indicate the slow progressor……. whereas the triangular dots indicate the rapid progressor. The lines connecting the circular and triangular dots represent each pair of twins” should be the legends to Fig 2.

**Response:** Many thanks for your valuable insights. As the reviewer advised, we clarified the title and legend of Figure 3 (figure 2 in the original manuscript).

Discussion:

Lines 183-186: The authors stated that they (their study) found that chronic illness and nutritional insufficiency might play a significant role in rapid kidney function decline in the general population.

This study did not demonstrate the above findings. Authors may need to re phrase this sentence.

**Response:** Thank you for pointing this out. This section was revised following your suggestion.

“In comparing baseline characteristics between groups, we found that several factors related to malnutrition, such as BMI, waist-hip ratio, body fat percentage, hemoglobin, fasting glucose, and triglyceride, showed significant differences in the high discordance group. In previous studies, body composition metrics, such as BMI, WHR, and total body fat percentage, were significantly correlated with individual nutritional status.” (Page 12, lines 194 to 198)
