# Supplementary material for: Risk factors associated with the discordance in kidney function decline rate in identical twins
Source: PLoS One. 2023 Apr 13;18(4):e0284154. doi: 10.1371/journal.pone.0284154 (PMC10101412; doi:10.1371/journal.pone.0284154)
Supplement: Attachment — Submitted filename: response_to_reviewers_R2.docx [file pone.0284154.s003.docx]

**Response to reviewers**

Reviewer #1: Thank you for inviting me to review the revised manuscript “Risk factors associated with the discordance in kidney function decline rate in identical twins.”

The authors have made requested corrections in the revised manuscript.

- Thank you for reviewing our manuscript. After revise according to your comments, our manuscript much improved.

Reviewer #2: Minor Revision:

1. Data Collection: Authors should include how of intra twin difference in annual eGFR decline and other variables (Demographic and clinical variables) were defined for this study.

- Thanks for the valuable comments. We described the methods how to calculate intra-twin difference of eGFR decline and other variables at the end of Data Collection paragraph.

In line #92-94,

“And intra-twin difference of eGFR decline rate was calculated by eGFR decline rate of one of the twin minus eGFR decline rate of the other of the twin. Intra-twin difference of other baseline clinical characteristics were also calculated by same manner.”

2. Study group Stratification: Line 95-99. Definition of slow and Rapid progressors and the example given by the authors was noted. However it will be nice for the author to let us know how the twins will be stratified in situations when the annual rate of eGFR decline in both twins are the same. For example, If the rate of eGFR decline in both twins is 1ml/min/1.73m2/yr OR rate of eGFR decline in both twins is 4ml/min/1.73m2/yr. In the 2 scenarios mentioned, how did the authors stratified into rapid / slow progressor when the annual rate of eGFR decline in both twins are the same.

- According to the reviewer’s comment, we appended additional clarification of group definition in the line #106-110 to avoid confusing the reader.

In line #106-110,

“These differences in eGFR decline rate within twins were classified regardless of their respective absolute eGFR decline rate. That is, even if both have a high eGFR decline rate (e.g. 7 ml/min/1.73m^2^/yr for both twins) or both have a low eGFR decline rate (e.g. 1 ml/min/1.73m^2^/yr for both twins), they were classified as the same group if the intra-twin difference of eGFR decline rate in each pair was the same.”

3. Result, Line 156: Please remove the phrase " which was similar to results in the paired t test.

- The phrase has been deleted according to the reviewer’s comment.

4. Table 1: Please change "Demographic characteristics" to " Demographics and Baseline Clinical Characteristics"

- We changed the sentence as recommended.

5. Discussion, line 225- 226. Please add reference to the sentence " Although, the exact mechanism...................based on previous studies". There should be a "comma" after the word "Although"

- We added comma after the word “Although”, and added references to the sentence.

6. Line 267: Better written as " Third, the cause of kidney dysfunction is not known because the cohort only has two time- point measurements (baseline and follow up) and lacks urinalysis.

- We changed the sentence according to the reviewer’s comment. Thank you for taking the time to review our manuscript.
