# Supplementary material for: Risk factors associated with the discordance in kidney function decline rate in identical twins
Source: PLoS One. 2023 Apr 13;18(4):e0284154. doi: 10.1371/journal.pone.0284154 (PMC10101412; doi:10.1371/journal.pone.0284154)
Supplement: Attachment — Submitted filename: response_to_reviewers_R3.docx [file pone.0284154.s004.docx]

Comments to the Author

Reviewer #2: Minor:

Authors are yet to answer one of my questions in the last review. The Authors' response to the question was for intra twin difference in eGFR decline and did not address my concern.

1. Study group Stratification: Definition of slow and Rapid progressors and the example given by the authors were noted i.e. when twins have an eGFR decline rate of 1 and 3 mL/min/1.73 m2 98 /yr, then one of the twins with an eGFR decline rate of 3 mL/min/1.73 m2 99 /yr is included in the rapid eGFR change group and the other twin with an eGFR decline rate of 1 mL/min/1.73 m2 100 /yr was included in the slow eGFR group.

My Question: How did the authors stratify the twins in situations where the annual rate of eGFR decline in both twins are the same. For example, If the rate of annual eGFR decline in each of the twin is 2ml/min/1.73m2/yr, how will you decide who will be the slow progressor/ rapid progressor group.

- We sincerely thank you for reviewing our manuscript. In our study, 8 pairs (2.4%) of twins showed exactly same eGFR change rates like as your example. For these twins, we randomly included individuals in each group, rather than manually selecting those with slow or rapid eGFR progression.

2. lines 92-94 should be written as "The annual eGFR decline rate was calculated using the formula: (eGFR at follow-up - eGFR at baseline)/follow-up duration (year). The intra-twin difference in eGFR decline rate was calculated by subtracting eGFR decline rate of the slow progressor twin from the eGFR decline rate of the other twin (who is the rapid progressor). Intra-twin difference of other baseline clinical characteristics were also calculated using the formula: (value in 'Slow progressor' - value in 'Rapid progressor')

- Thank you so much for correcting my English. I have changed the mentioned sentence as you suggested.

Line 145: Slow progressor " not low progressor"

- I fixed a typo in the subheading. Thanks for pointing it out.
