# Supplementary material for: Risk factors associated with the discordance in kidney function decline rate in identical twins
Source: PLoS One. 2023 Apr 13;18(4):e0284154. doi: 10.1371/journal.pone.0284154 (PMC10101412; doi:10.1371/journal.pone.0284154)
Supplement: Attachment — Submitted filename: response_to_reviewers_R4.docx [file pone.0284154.s005.docx]

Comments to the Author

Reviewer #2: Thank you for addressing the concerns on study group classification. However the authors are yet to include this in the method section of this manuscript. Please, I suggest you kindly make amends.

STUDY GROUP CLASSIFICATION Page 6:

1. Line 98-99: I suggest you re write the first sentence as

"All twins were assigned to either the rapid or slow eGFR change groups based on their annual eGFR decline rate"

- Thank you for your time to review and correct our manuscript. We changed the sentence as you recommended.

2. I suggest that the sentence " For pair of twins with the same annual eGFR decline rate, we randomly included each twin in each group, rather than manually selecting those with slow or rapid eGFR progression based on the aforementioned criteria." should come after the sentence "All twins were included in the relatively rapid or slow...............................in slow eGFR change" (lines 100-104)

- We added the sentence on line 102-104.

3. Delete the sentence "That is, even if both have a high eGFR decline rate (e.g. 7 ml/min/1.73m2/yr for both twins) or both have a low eGFR decline rate (e.g. 1 ml/min/1.73m2/yr for both twins), they were classified as the same group if the intra-twin difference of eGFR decline rate in each pair was the same. (lines 1111-113)

- We deleted it.
